# Association between a four-locus gene model including *IL13*, *IL4*, *FCER1B*, and *ADRB2* and asthma outcomes

**DOI:** 10.3389/fped.2026.1815201

**Published:** 2026-05-07

**Authors:** Shasha Bai, Linlin Qin, Tingting Zhou, Shaomin Chen, Fen Yang, Li Hua, Yixiao Bao

**Affiliations:** 1 Department of Pediatric Pulmonology, Xinhua Hospital Affiliated to Shanghai Jiao Tong University School of Medicine, Shanghai, China; 2 Thoracic Surgery, Shanghai Fourth People’s Hospital, Shanghai, China; 3 Department of Pulmonology, Xinhua Hospital Affiliated to Shanghai Jiao Tong University School of Medicine, Shanghai, China; 4 Shanghai Tonxin Clinic, Shanghai, China

**Keywords:** asthma, course, gene, model, outcome

## Abstract

**Background:**

This study aimed to explore the association between a four-locus gene model and asthma outcomes.

**Methods:**

Four single-nucleotide polymorphisms were genotyped in 139 patients with different asthma outcomes (clinical remission, relapse, or persistence). The high-risk and low-risk genotypes for asthma were evaluated according to the different genotypes that they carried. Differences in asthma outcomes and the factors affecting asthma prognosis were analyzed.

**Results:**

More patients with high-risk genotypes had severe asthma attacks than those with low-risk genotypes. Compared with patients in asthma clinical remission, patients with severe asthma attacks were at risk for asthma relapse and asthma persistence. In addition, smoking was a risk factor for asthma persistence. The older the age of asthma onset, the more likely it was to be persistent. More patients in the high-risk genotype group had an age of asthma onset younger than 6 years old and an asthma course longer than 4 years than in the low-risk genotype group, regardless of the confounding factors.

**Conclusions:**

The patients with high-risk genotypes not only had more severe asthma attacks and a younger age of asthma onset but also had a longer course, which suggested that the gene model could serve as a tool for asthma prognosis assessment with moderate utility.

## Introduction

In recent years, with the further understanding of and research on asthma, the prognosis and influencing factors of asthma have become the subjects of numerous studies. Asthma mostly occurs in preschool-aged children, and changes in airway structure and lung function damage resulting from asthma may begin earlier (1). The wheezing phenotype in preschool-aged children can be divided into the following: (1) early transient wheezing: children whose wheezing disappears before 3 years old; (2) early onset persistent wheezing: children who develop the disease before the age of 3 generally experience wheezing symptoms that persist until school age, with some children still experiencing symptoms at the age of 12; and (3) delayed wheezing or asthma: children with typical characteristic backgrounds often experience asthma symptoms that persist into adulthood (2). These different wheezing phenotypes suggest the necessity of paying attention to asthma outcomes. When children grow older, their immune function and body resistance improve, and some children with asthma experience symptom relief during puberty and adulthood. Studies have reported asthma relief rates ranging from 20% to 70% from childhood to adulthood (3–5).

Currently, there are few studies on childhood asthma outcomes. Prospective studies in multiple large birth cohorts have shown that the factors affecting the relief of childhood asthma are the following: (1) gender: asthma relief or self-healing mostly occurs in male patients, while being female is a risk factor for asthma lasting into adulthood (6); (2) asthma severity: a follow-up birth cohort study found that childhood asthma severity determines prognosis and outcomes, and they proved that the more severe the childhood asthma is, the more likely it is to persist into adulthood (7); (3) age of onset: there is controversy over the impact of onset age on the outcome of asthma. Some studies suggest that the younger the age of asthma onset, the greater the probability of recurrence and the more likely it is to persist into adulthood (8). However, others indicate that the earlier the onset of asthma, the easier it is to alleviate it (9); (4) manifestations: multiple sensitizations and early sensitization are risk factors for persistent childhood asthma; (5) combination of allergic diseases: most children with asthma have concurrent allergic diseases, such as allergic rhinitis and allergic dermatitis. Studies suggest that the accompanying allergic complications are risk factors for difficult-to-treat childhood asthma (10); (6) airway hyper responsiveness: airway hyperresponsiveness and its severity are closely related to childhood asthma prognosis (11); (7) mother’s allergy history: Dutch researchers followed up an asthma cohort for 39 years and found that a positive mother’s allergy history is one of the risk factors for childhood asthma to persist into adulthood (6); and (8) genetics: some studies suggest that genetics can increase the risk of persistent asthma, and polygenic genetic risks can predict the occurrence and progression of childhood asthma (12). There is limited understanding of which patients experience remission of asthma in adulthood and how it is related to genetic factors in China. Moreover, the natural course and treatment time of patients with asthma are also challenges faced by clinical doctors. Currently, there is no effective method to predict the course of patients with asthma. We suggest the hypothesis that a gene model can be used to predict the course of asthma in patients.

Hence, our study recruited patients with asthma of different onset ages and classified their asthma status based on their asthma development trend. The differences in asthma onset age and duration between different risk genotypes based on our model were compared. The aim was to elucidate the impact of genetic factors on the prognosis of childhood asthma in Han patients and provide a scientific basis for evaluating asthma prognosis.

## Methods

### Study population

Our study recruited 139 patients with asthma from the Respiratory Clinic in Xinhua Hospital Affiliated to Shanghai Jiaotong University School of Medicine. The study inclusion criteria consisted of the following: (1) adult patients diagnosed with bronchial asthma by doctors using the diagnostic criteria from the 2016 bronchial asthma prevention and treatment guidelines by the Asthma Group of the Respiratory Disease Branch of the Chinese Medical Association (13); (2) individuals with a history of asthma but currently in remission; and (3) all participants were of Han ethnicity, aged 18–65, and had signed an informed consent. The exclusion criteria were as follows: (1) individuals with mental illness; (2) patients with asthma combined with consciousness disorders; and (3) patients with asthma combined with chronic obstructive pulmonary disease. We collected asthma-related information from the included patients, including the age of asthma onset, current asthma status, clinical remission of asthma, recurrence and persistence of asthma after remission, severity of asthma attacks, personal allergy history, family history, and occupational risk factors. The study was approved by the ethics committee of the hospital (No. XHEC -D - 2016–393) and was conducted according to the principles of the Declaration of Helsinki.

### Genotyping and grouping

The genomic DNA of the four genes was extracted from the buccal mucosa using a DNA extraction kit (Thermo Fisher Scientific, Waltham, MA, USA). The genotypes of the four gene loci were obtained using a multiplex polymerase chain reaction-restriction fragment length polymorphism assay (PCR-RFLP). The PCR cycling conditions were as follows: 95°C for 2 min; 45 cycles at 95°C for 30 s, 56°C for 30 s, and 72°C for 60 s; and finally, 72°C for 5 min. The conditions for the shrimp alkaline phosphatase (SAP) enzyme digestion reaction were 37°C for 40 min and then termination at 85°C for 5 min. The conditions used for iPLEX were 95°C for 30 s; 5 inner cycles at 52°C for 5 s and at 85°C for 5 s; and 40 outer cycles at 94°C for 5 s, 52°C for 5 s, and 85°C for 5 s. We then used matrix-assisted laser desorption ionization time-of-flight mass spectrometry to discriminate between the four single-nucleotide polymorphisms (SNPs) (14).

In our study, the different risk genotypes for asthma were discriminated according to the number of risk allele homozygotes. The control group comprised patients with no risk homozygotes (i.e., IL13 rs20541 AA or GA, IL4 rs2243250 CC or TC, ADRB2 rs1042713 GG or AG, and FCER1B rs569108 AA or AG). Those with different genotype combinations who had a significantly higher risk of asthma (if *P* < 0.05 and OR > 1) than the reference group were defined as being at higher genetic risk for asthma. The others were defined as being at lower genetic risk for asthma. The results for the different genotype combinations are shown in Table 1 (14).

**Table 1:** High- and low-risk asthma genotypes according to the four genotypes.

Group	Genotype			
	IL13 rs20541	IL4 rs2243250	ADRB2 rs1042713	FCER1B rs569108
Low-risk genotypes	AA or GA	CC or TC	GG or AG	AA or AG
	AA or GA	CC or TC	GG or AG	GG
	AA or GA	CC or TC	AA	AA or AG
	AA or GA	TT	GG or AG	AA or AG
	GG	CC or TC	GG or AG	AA or AG
	AA or GA	CC or TC	AA	GG
	GG	CC or TC	GG or AG	GG
High-risk genotypes	AA or GA	TT	GG or AG	GG
	AA or GA	TT	AA	AA or AG
	GG	CC or TC	AA	AA or AG
	GG	TT	GG or AG	AA or AG
	AA or GA	TT	AA	GG
	GG	CC or TC	AA	GG
	GG	TT	GG or AG	GG
	GG	TT	AA	AA or AG
	GG	TT	AA	GG

### Evaluation of different asthma states

In this study, the asthma status of patients was divided into clinical remission, recurrence after remission, or persistent asthma. Currently, there is no unified standard for the clinical remission of asthma. A study in Taiwan defined the absence of wheezing attacks in patients for 3 years prior to the follow-up cut-off as clinical remission of asthma (15). In the study by Alberta et al., clinical remission of asthma was defined as simultaneously meeting the following three criteria: (a) no asthma symptom onset, (b) no use of anti-asthma drugs, and (c) no evidence of airflow restriction [lung function test: forced expiratory volume in 1 s/forced vital capacity (FEV1/FVC) ≥ 80%], as of the year before the follow-up cutoff (11). Complete remission of asthma requires a negative bronchial provocation test in addition to meeting the conditions for clinical remission. According to this standard, very few people will experience complete remission of asthma. In several studies, clinical remission was defined as the absence of asthma attacks in the 2 years prior to follow-up and at least 1 year without the use of anti-asthma drugs, resulting in reasonable results (16, 17). In addition, our previous studies on the outcome of wheezing in young children have also used the standard of no asthma symptoms in the 2 years prior to the follow-up cut-off point and no use of anti-asthma drugs for at least 1 year as clinical relief for wheezing (18). Therefore, we will continue to use this method in our study. Asthma relapse after remission refers to the recurrence of asthma symptoms after at least 2 years of remission, or the use of asthma control drugs within the past year. Finally, persistent asthma refers to the absence of any asthma attacks for 2 years.

### Statistical analysis

We used SPSS 20.0 (IBM, USA) to conduct the statistical analyses in this study. The quantitative data are expressed as mean ± standard deviation. Comparison of means between two samples was conducted using Student’s *t*-test or rank sum test, and Pearson *χ*^2^ test or Fisher’s exact probability method was used to compare the differences in asthma status and duration between the patients with asthma with high-risk and low-risk genotypes. Logistic regression analysis was performed to elucidate the influencing factors for different asthma states in patients, and the odds ratio and 95% confidence interval were calculated. All the tests were double-sided tests, and the test level was *α* = 0.05, with *P* < 0.05 indicating a statistically significant difference.

## Results

### Basic characteristics of the patients with asthma with high-risk and low-risk genotypes

In our study, 139 patients of the Han ethnicity were enrolled. The combinations of different asthma risk alleles carried by these patients were used to classify high- and low-risk asthma genotypes. The results showed that 62 patients had high-risk asthma genotypes and 77 had low-risk asthma genotypes. Women accounted for 53.23% of the high-risk genotype group, which was higher than that in the low-risk genotype group. There was no statistically significant difference between the two groups. In addition, 56.45% of the patients with high-risk genotypes experienced severe asthma attacks, which was higher than those with low-risk genotypes. This difference between the two groups was statistically significant. There were 36 patients with clinical remission of asthma, with a remission rate of 25.90%. Patients with recurrent and persistent asthma after remission accounted for 31.65% and 42.45%, respectively. There were no statistically significant differences in age, allergic rhinitis, eczema or atopic dermatitis, positive family allergy history, asthma history in first-degree relatives, smoking history, occupational risk factors, and different asthma states (Table 2).

**Table 2:** The distribution of basic characteristics in the patients with asthma and high-risk and low-risk genotypes.

Variable	High-risk genotype group	Low-risk genotype group	*P*-value
No.	62	77	NA
Female (%)	33 (53.23%)	37 (48.05%)	0.54
Age, years $\left(\bar{x}\pm s\right)$	35.18 ± 1.93	36.62 ± 1.96	0.60
Severe asthma attacks (%)	35 (56.45%)	29 (40.23%)	**0**.**027**
Allergic rhinitis (%)	44 (70.97%)	63 (81.82%)	0.13
Eczema/atopic dermatitis (%)	35 (56.45%)	36 (46.75%)	0.26
Family allergy^a^ (%)	29 (46.77%)	40 (51.95%)	0.54
Asthma in first-degree relatives (%)	11 (17.74%)	18 (23.38%)	0.42
Smoking (%)	8 (12.90%)	16 (20.78%)	0.22
Occupational risk factors^b^ (%)	11 (17.74%)	13 (16.88%)	0.89
Clinical remission of asthma (%)	14 (38.89%)	22 (61.11%)	
Recurrence of asthma after remission (%)	24 (54.55%)	20 (45.45%)	0.16^c^
Persistence of asthma (%)	24 (40.68%)	35 (59.32%)	0.86^c^

a Positive history of family allergy was defined as the following. The patient’s first- and/or second-degree relatives met at least one of the following four criteria: (1) eczema or atopic dermatitis diagnosed by a doctor; (2) doctor-diagnosed allergic rhinitis; (3) bronchial asthma diagnosed by a doctor; and (4) food allergy diagnosed by a doctor (11).

b Occupational risk factors refer to the following criteria. Patients must meet at least one of the following occupational risk factors: (1) occupational exposure to harmful gases; (2) occupational exposure to dust; and (3) frequent exposure to biofuel smoke.

c Using clinical remission of asthma as a reference.

The bold values are *P* < 0.05, indicating a statistically significant difference.

### Analysis of influencing factors for different asthma states

We then used unordered multiclass logistic regression to analyze the factors affecting different asthma states in the patients. We found that compared with clinically relieved patients with asthma, severe asthma attacks are a risk factor for asthma recurrence and persistence after remission. The older the age of asthma onset, the more likely it was to be persistent, but it had no significant effect on asthma recurrence after remission. In addition, the patients with a positive smoking history were more likely to experience persistent asthma. Clinical remission of asthma was not significantly affected by gender, age, different asthma risk types, allergic rhinitis, eczema, family allergy history, asthma history in first-degree relatives, and occupational risk factors (Table 3).

**Table 3:** Analysis of influencing factors for different asthma states.

Variable	Recurrence vs. clinical remission^a^		Persistence vs. clinical remission^a^	
	*P*-value	OR (95% CI)	*P*-value	OR (95% CI)
Female vs. male	0.49	1.37 (0.57–3.32)	0.44	1.38 (0.60–3.18)
Age	0.77	1.01 (0.98–1.03)	0.38	1.01 (0.99–1.04)
Asthma genotype				
High-risk vs. low-risk	0.16	1.89 (0.77–4.61)	0.86	1.07 (0.46–2.51)
Severe asthma attacks				
Positive vs. negative	**0**.**021**	3.19 (1.19–8.55)	**0**.**024**	2.95 (1.16–7.52)
Asthma onset age	0.19	1.03 (0.99–1.08)	**0**.**001**	1.07 (1.03–1.12)
Allergic rhinitis				
Positive vs. negative	0.78	1.15 (0.42–3.13)	0.30	1.68 (0.63–4.46)
Eczema				
Positive vs. negative	0.27	1.64 (0.68–4.00)	0.55	1.29 (0.56–2.98)
Family allergy				
Positive vs. negative	0.77	1.14 (0.47–2.76)	0.36	1.48 (0.64–3.41)
Asthma in first-degree relatives				
Positive vs. negative	0.37	1.67 (0.55–5.05)	0.66	1.28 (0.43–3.76)
Smoking				
Positive vs. negative	0.07	2.78 (0.91–8.50)	**0**.**029**	3.27 (1.13–9.44)
Occupational risk factors				
Positive vs. negative	0.44	1.59 (0.48–5.26)	0.69	1.27(0.40–4.05)

OR, odds ratio; CI, confidence interval.

a We used unordered multiclass logistic regression analysis, with clinical remission of asthma as the reference.

The bold values are *P* < 0.05, indicating a statistically significant difference.

### Comparison of the age of asthma onset between patients with high-risk and low-risk genotypes

We conducted a stratified analysis of the age of asthma onset and found that compared to patients with asthma with low-risk genotypes, more patients with asthma with high-risk genotypes developed symptoms before 6 years old. After adjusting for confounding factors such as gender and age, this result still showed statistical differences (Table 4).

**Table 4:** Comparison of the age of asthma onset between patients with high-risk and low-risk genotypes.

Onset age	High-risk	Low-risk	Uncorrected		Corrected^a^	
			*P*	OR (95% CI)	*P*	OR (95% CI)
<6 years old	36 (53.73%)	31 (46.27%)	0.037	2.06 (1.04–4.05)	0.047	2.05 (1.01–4.17)
≥6 years old	26 (36.11%)	46 (63.89%)				

OR, odds ratio; CI, confidence interval.

a Logistic regression correction for confounding factors, i.e., gender and age.

### Comparison of the course between patients with high-risk and low-risk genotypes

We found that the average asthma course in the patients with high-risk genotypes was 19.43 years, while in the patients with low-risk genotypes, it was 14.21 years. While the former was higher than the latter, there was no significant statistical difference between the two groups. We further stratified the course of asthma and found that 79.03% of patients with asthma with high-risk genotypes had a course longer than 4 years, which was higher than the proportion of patients with asthma with a course ≤4 years. There was a statistically significant difference between the two groups, and after adjusting for confounding factors, such as gender and age, this result was still statistically significant (Table 5).

**Table 5:** Comparison of the course between patients with high-risk and low-risk genotypes.

Course	High-risk	Low-risk	Uncorrected		Corrected^a^	
			*P*	OR (95% CI)	*P*	OR(95% CI)
$\left(\bar{x}\pm s\right)$	19.43 ± 16.23	14.21 ± 15.68	0.057	NA	0.065	NA
≤4 years	13 (20.97%)	29 (37.66%)	0.033	2.28 (1.06–4.90)	0.045	2.22 (1.02–4.85)
>4 years	49 (79.03%)	48 (62.34%)				

OR, odds ratio; CI, confidence interval.

a Logistic regression correction for confounding factors, i.e., gender and age.

## Discussion

Asthma is an incurable chronic respiratory disease, and its outcome and prognosis remain challenges in scientific research. With research on complex asthma phenotypes, our understanding of asthma is no longer limited to controlling the condition, as uncovering the risk factors that affect the persistence or recurrence of asthma and intervening in the occurrence and development of asthma are particularly important. The development of asthma follows a certain natural trend, referred to as the “natural course” (19). There are individual differences in the natural course of each patient, so it is difficult for doctors to determine how long each patient’s natural course is. The use of anti-asthma drugs can only control the condition of patients with asthma and cannot improve the natural course of the disease. The majority of asthma cases begin before school age, and some children may experience relief from asthma symptoms as they age and when their immune function changes. At present, research on asthma outcomes is mostly based on prospective follow-up studies in large birth cohorts. Due to the lack of a unified definition of asthma remission and persistence in these studies and the different follow-up times, reports on asthma remission rates vary. A study in a multiethnic population of patients with mild-to-moderate persistent childhood asthma showed a complete remission rate of 26% (11). Research by Heldin et al. showed that approximately three-quarters of school-aged children experience asthma relief by mid-adulthood, which refers to the relief of clinical symptoms of asthma (20). In addition, a follow-up study on asthma in children from birth to the age of 26 found that the wheezing relief rate in children was 27.4%, and wheezing symptoms persisted into adulthood in more than 1/4 of children (4). In our study, the asthma clinical remission rate was 25.90% when using the criteria of no asthma symptoms in the 2 years prior to the follow-up cut-off point and no anti-asthma drugs used for at least 1 year.

This study identified the following three influencing factors affecting asthma outcomes: (1) asthma onset age, i.e., the older the age of asthma onset, the more likely it is for the patient to experience persistent asthma; (2) severity of asthma attacks, i.e., those who experience severe attacks are less likely to experience clinical remission; and (3) smoking history, i.e., a positive smoking history is a risk factor for persistent asthma. These findings are consistent with the results of multiple international studies on the prognostic factors of asthma. In addition, we also found that more patients with high-risk genotypes had more severe asthma attacks than those with low-risk genotypes, which indicates an association between the asthma susceptibility gene loci included in the gene model and severe asthma attacks (21–23). Severe asthma attacks are also a risk factor for asthma recurrence and persistence, so we speculate that the model can indirectly reflect these asthma outcomes. This study analyzed the onset age of patients with different asthma risk types and found that patients with asthma with high-risk genotypes had an earlier onset age. More than half of the patients with asthma with high-risk genotypes had already developed asthma before 6 years old, which is consistent with the fact that the majority of asthma cases occur in early childhood, especially preschool age. Furthermore, a subgroup analysis found that more patients with asthma with high-risk genotypes had a disease course of more than 4 years, indicating that the disease course of patients with asthma with high-risk genotypes is longer.

However, several study limitations should be acknowledged. First, the relatively small sample size in this study may have led to biased results. In addition, confounding factors were not adequately controlled, such as socioeconomic status, environmental exposures, adherence to medication, or access to healthcare. Therefore, subsequent studies should increase the sample size to enable more comprehensive analysis and further clarify the impact of this genetic model on asthma prognosis.

In summary, the study indicates that this four-locus gene model is related to the onset age, with patients with asthma with high-risk genotypes having an earlier onset age. In addition, compared with the patients with asthma with low-risk genotypes, the patients with asthma with high-risk genotypes had more severe asthma attacks and longer disease duration. Age of onset, severity of asthma, and smoking history are risk factors that affect asthma prognosis. These results fully demonstrate the association of the model with disease prognosis, providing strong evidence for clinical doctors to solve the treatment course problem in childhood asthma.

## Conclusions

We found that the patients carrying high-risk genotypes exhibited more severe asthma attacks, an earlier age of onset, and a longer disease course. This research demonstrated that the model can be used to evaluate asthma prognosis to a certain extent.

## Data Availability

The data that support the findings are available from the corresponding author upon reasonable request.
